# Predicting New Graduate Nurses' Retention during Transition Using Decision Tree Methods: A Longitudinal Study

**DOI:** 10.1155/2024/4687000

**Published:** 2024-05-28

**Authors:** Taewha Lee, Yea Seul Yoon, Yoonjung Ji

**Affiliations:** ^1^Mo-Im Kim Nursing Research Institute, College of Nursing, Yonsei University, Seoul, Republic of Korea; ^2^College of Nursing, CHA University, Pocheon, Republic of Korea; ^3^Brain Korea 21 FOUR Project, College of Nursing, Yonsei University, Seoul, Republic of Korea

## Abstract

**Background:**

Although retaining new nurses is imperative for the future of the nursing profession, it remains a challenging task in the healthcare industry. Understanding the career journey of new graduates as they transition from students to nurses is vital. However, longitudinal studies investigating the factors influencing retention during this period are lacking.

**Aim:**

The aim of this study is to identify the influencing factors and develop a longitudinal prediction model for new graduate nurse retention.

**Methods:**

A secondary data analysis was conducted using the New Nurse e-Cohort Study dataset from two survey periods, November–December 2020 and February–March 2022. The participants were categorized into either retention or turnover groups based on their turnover experiences. A decision tree based on classification and regression tree (CART) analysis was utilized.

**Results:**

Of the total 586 participants, 463 (79%) were in the retention group. The CART model highlighted that new nurses' retention was significantly associated with younger age, higher readiness for practice (clinical problem-solving) during the nursing program, lower transition shock (such as confusion in professional values, loss of social support, and conflicts between theory and practice), and a higher person-environment fit (person-job fit). The predictive accuracy of the CART model was 79.7%.

**Conclusion:**

To retain new nurses, nursing educators and hospital managers should collaborate to prepare nursing students for actual practice, offer support during organizational socialization, and foster healthy professional values for competence in the workplace. *Implications for Nursing Management*. Transforming the educational strategies of nursing programs and hospital management policies is imperative to ultimately enhance the retention of new graduate nurses.

## 1. Introduction

Accounting for over 50% of the global health workforce, there are approximately 28 million nurses worldwide [1]. New and experienced nurses are essential to the profession's future, but retaining them within the healthcare industry continues to be challenging. As a result, by 2030, there is expected to be a shortage of 5.9 million nurses [1], leading to a vicious cycle of far-reaching consequences on patient ratios, staff dissatisfaction, occupational stress, burnout, and staff retention issues, ultimately compromising patient safety and the quality of care [2, 3]. Turnover is especially a problem among new graduate nurses; up to 30% and 57% leave within their first year and by the end of their second year, respectively [4]. New nurse turnover represents a loss of human and financial resources for the healthcare workforce, which is particularly problematic given the prevailing global shortage of nurses [5]. Preventing the exodus of new nurses is crucial; specifically, it requires developing an understanding of how new nurses adapt to new roles, from students to nurses [6], the reasons for the exodus, and multifaceted solutions to prevent it.

The first year of employment is the period for nursing students to grow and adapt as competent professional nurses. The successful transition to practice is an important factor that determines the retention of new nurses [7]. During the transition, new nurses confront numerous challenges, such as a theory-practice gap, overwhelming workload, difficulties in interpersonal adaptation with coworkers, confusion in professional identities, lack of nursing competency [8], and work-life imbalance [8, 9].

Previous studies have contributed to understanding the diverse factors related to the reasons for new nurse turnover and recommended policy strategies to retain them in practice. New nurses' turnover reasons appear to increase in a poor working environment [10, 11] and when nurses experience workplace bullying [12] or violence [13]. In addition, low resilience with less empowerment [11], low job satisfaction [13, 14], and high job stress [15] provoke high turnover intention. However, as previous studies focused on the relationship between these factors and turnover intention rather than actual turnover, there is insufficient evidence to determine whether the reported factors really predict nurse turnover in practice settings.

Focusing on the factors leading to the retention of new nurses is more effective than identifying the factors involved in turnover; a positive approach not only controls problems but also goes beyond elimination and leads to a healthy work environment and better work life quality in the nursing profession [16]. In addition, identifying and enhancing retention factors can create a positive organizational environment that encourages new nurses to continue to work, leading to improved quality of nursing and patient safety [17]. Brook et al.'s systematic review [18] indicated that interventions aimed at retaining new nurses, including residency programs [19] and transition programs [20], have been relatively successful. However, the duration for intervention effects to manifest, ranging from 27 to 52 weeks, is relatively long, and sustaining the intervention during this period requires additional staffing and resources [18]. This raises doubts about the feasibility of implementation in countries with limited resources and support. In fact, out of the 53 studies included in the abovementioned systematic review, 52 were conducted in high-income countries such as the USA, Australia, the UK, and Canada [18].

Other descriptive studies have identified several organizational and personal factors that positively affect the retention intention of new nurses, including organizational culture, empowering leadership, organizational socialization [21], the meaning of work, organizational commitment, professional self-image [22], clinical competency, and grit [23]. To ascertain the factors influencing the actual retention of nurses, it is necessary to confirm whether retention intention translates to actual retention. Yet, similar to studies on nurse turnover, most nurse retention studies have identified a relationship between relevant factors and retention intention, not actual retention [24, 25]; thus, whether the factors affecting retention intention also affect retention is unclear. Therefore, to address the global nurse shortage, it is imperative to identify universally applicable factors influencing the retention of new nurses and formulate appropriate strategies [26].

Despite knowledge of their importance, the factors influencing the retention of new nurses have rarely been studied longitudinally. Most previous studies have focused on the experiences of new nurses after employment using cross-sectional data [5, 8, 27], thereby limiting their predictive abilities. To understand the retention factors of new graduate nurses, their transition from students to nurses should be examined using longitudinal data. A previous study reported in-depth results by longitudinally analyzing the factors affecting the transition shock caused by the conversion of students into practicing new nurses; however, whether these factors eventually lead to the retention of new nurses has not been confirmed [6].

**Figure 1:**
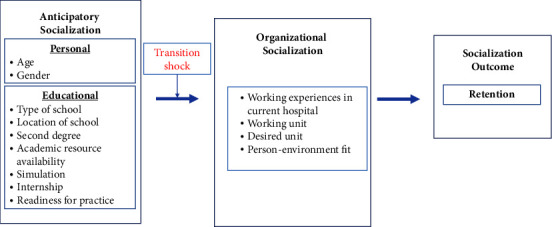
Conceptual framework to predict retention among new nurses based on Scott's model (2008) of the transition of new graduate nurses into the workplace.

Therefore, we aimed to identify the factors influencing new graduate nurse retention, utilizing decision tree analysis to uncover the following two key aspects: the anticipatory socialization factors of nursing students and the organizational socialization factors of new graduate nurses based on Scott's 2008 model of new graduate nurses transitioning into the workplace [28] (Figure 1).

### 1.1. Conceptual Framework

The model proposes that anticipatory and organizational socialization experiences concurrently influence the successful transition of new nurses into the workplace [28]. According to this model, the transition of new nurses into the workplace is characterized by the following three phases of progression: anticipatory socialization, organizational socialization, and socialization outcomes. Anticipatory socialization refers to the educational or personal characteristics of a student who is preparing to start work as a nurse, such as age, gender, and educational preparation. Organizational socialization refers to all experiences that new nurses have after beginning work, such as work stressors, preceptorship, orientation, and person-environment fit. Socialization outcomes refer to negative or positive outcomes depending on new nurses' perceptions of practice, the organizational education program provided, personal conditions, and work environment. Dissatisfaction with work and career can lead to turnover, while satisfaction leads to retention.

## 2. Materials and Methods

### 2.1. Study Design and Data Source

**Figure 2:**
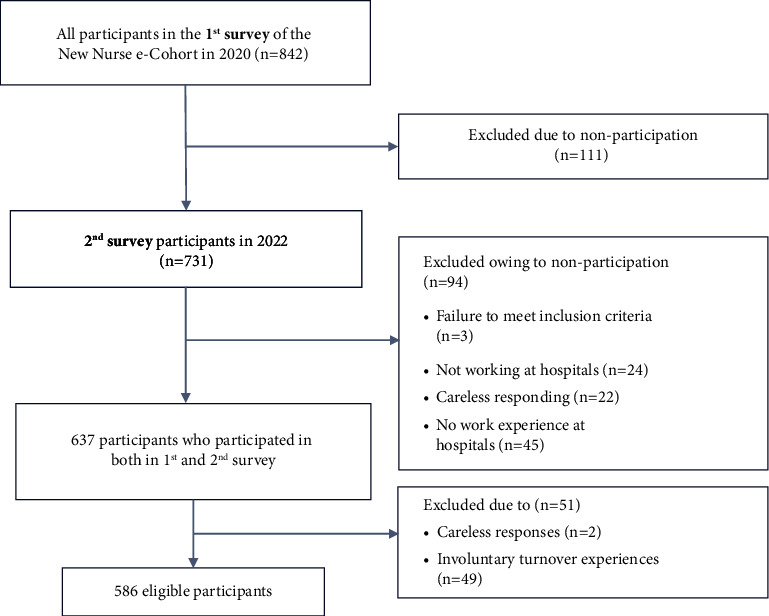
Flow diagram of participant selection.

A longitudinal study was conducted using the datasets of the first and second surveys of the New Nurse e-Cohort Study [29] in South Korea (Figure 2). The New Nurse e-Cohort Study is a longitudinal panel study that tracks senior nursing students for three years to identify factors influencing the successful transition to new nurses. The panel was constructed in 2020, with the second and third surveys conducted in 2022 and 2023, respectively. The data for the first survey were collected from November to December 2020, when all participants were senior nursing students. The data for the second survey were collected from February to March 2022, the year after the participants had graduated. The second survey was conducted when participants had experience working as new graduate nurses.

### 2.2. Study Sample

We included 637 (75.6%) participants out of 842 senior nursing students aged 20–29 who were scheduled to graduate in 2021 and take the national nurse licensure examination that year and who reported having worked in hospitals at the time of the second survey in 2022. Among them, 586 were included in the final analysis after excluding 49 with involuntary turnover experiences and two with careless responses. In the original study [29], additional consent for secondary data analysis was requested at the time of initial data collection, and only the data of participants who agreed to secondary data analysis were used in this study.

### 2.3. Measurements

Factors affecting retention were allocated to one of two categories based on Scott's model [28].

### 2.4. Anticipatory Socialization

  Personal factors included participants' demographic characteristics (age and gender).
  Educational factors included information related to the participants' prelicensure nursing education, including the type and location of the school, second degree program, internship experience, availability of academic resources, and simulation education. Academic resource availability was measured by asking one yes/no question as follows: “have you been able to use any materials from the school library?” Simulation education was measured by asking another yes/no question as follows: “have you received simulation education?”
  Readiness for practice was assessed using the comfort and confidence section of the Casey-Fink Readiness for Practice Survey [8]. This self-report questionnaire consists of 20 items divided into the following four subscales: clinical problem-solving, learning techniques, professional identity, and trials and tribulations. Participants rated each item on a 4-point Likert scale, ranging from 1 (strongly disagree) to 4 (strongly agree). The average score across all items is used as the scale score, where a higher score indicates greater readiness for practical application. Cronbach's *α* was 0.69 at the time of the instrument's development [8] and 0.83 in our study.

### 2.5. Organizational Socialization

Organizational socialization included information related to the first job experience at a hospital after graduation, including work characteristics (number of months worked, working unit, placement in desired unit, and number of preceptors), transition shock, and person-environment fit.

#### 2.5.1. Transition Shock

Transition shock was measured using the Transition Shock Scale for newly graduated nurses [30]. This 18-item self-report questionnaire contains the following six subscales: conflict between theory and practice, overwhelming workload, loss of social support, shrinking relationships with coworkers, confusion in professional nursing values, and incongruity in work and personal life. The items are rated on a 4-point Likert scale ranging from 1 (strongly disagree) to 4 (strongly agree). The mean of all items is considered the scale score, with a higher score indicating higher transition shock. Cronbach's *α* was 0.89 at the time of the instrument's development [30] and 0.89 in our study.

#### 2.5.2. Person-Environment Fit

Person-environment fit was measured using person-job and person-organization fit perception scale [31]. This eight-item self-report questionnaire contains the following two subscales: person-job and person-organization fit. The items are rated on a 5-point Likert scale ranging from 1 (very little) to 5 (very large extent). The mean of all items is considered the scale score, with a higher score indicating better person-environment fit. Cronbach's *α* was 0.86 at the time of the instrument's development [31] and 0.89 in our study.

### 2.6. Socialization Outcome

#### 2.6.1. Retention

Retention of new graduate nurses was assessed using a self-report questionnaire developed by our research team; during the second survey, participants were asked if they had experienced a voluntary change in workplaces since the first survey. Depending on their answers, the participants were divided into retention and turnover groups.

### 2.7. Ethical Considerations

This study adhered to the guidelines outlined in the Declaration of Helsinki [32], and approval to conduct a secondary data analysis was obtained from the Institutional Review Board of Yonsei University Medical Center (approval no: 4-2023-0854). All data were anonymized before being shared with the research team.

### 2.8. Data Analysis

The descriptive characteristics of the sample were computed using means and standard deviations for continuous variables and frequencies for categorical variables. Differences between the retention and turnover groups were assessed by *t*-tests and *χ*^2^ tests. Statistically significant variables (*p* < 0.05) in the univariate analysis were entered into the multivariate logistic regression analysis to develop the decision tree model. Decision tree analysis is a machine-learning technique [33] that provides a sequential and hierarchical path to an outcome variable from independent variables and demonstrates the retention probability given the specific values of the independent variables in the path. A decision tree based on classification and regression tree (CART) analysis was used to investigate the factors related to the retention of new graduate nurses. CART is an explanatory technique used mainly to identify data structures by splitting the data into segments that are as homogeneous as possible with respect to the dependent variables [34]. A classification model was constructed from the entire sample to create the largest tree structure with a minimum of 100 and 15 parent code and child node samples, respectively. K-fold cross-validation, with the value set in a 10-fold cross-validation sampling method, was used to address possible overfitting. The area under the receiver operating characteristic curve was calculated to assess the predictive performance of the decision tree for the retention of new graduate nurses. Statistical analyses were performed using SPSS Statistics for Windows version 26 (IBM Corp., Armonk, NY, USA).

## 3. Results

### 3.1. Participant Characteristics

Data from the 586 participants are presented in Table 1. A total of 463 participants (79.0%) had no job turnover experience since starting their first nursing job at a hospital. Regarding anticipatory socialization factors, the mean age of the participants was 24.8 ± 1.3 years, and the majority (91.0%) was women. Overall, 82.6% of the participants had graduated from university and most attended nursing schools located in the capital area (52.6%). Only 4.3% of the participants had achieved a second degree, 68.3% had academic resources available, and 97.6% had experience with simulation education. Of the participants, 91.3% had not undergone an internship at a hospital when they were nursing students. The mean readiness for practice score at the time of graduation was 2.86 (SD = 0.31) on a 4-point scale. Among the subscales, “professional identity” (*M* = 2.94, SD = 0.43) was highest, followed by “learning technique” (*M* = 2.92, SD = 0.55), “clinical problem-solving” (*M* = 2.88, SD = 0.77), and “trials and tribulations” (*M* = 2.73, SD = 0.35).

In terms of socialization factors, the mean working experience at the current hospital was 8.3 (SD = 3.9) months. Of the participants, 54.8% worked in a general ward and 57.4% were assigned to the unit they desired. The highest number of participants (62.7%) answered that one instructor had educated them during the new nurse orientation period. The mean score of transition shock was 2.82 (SD = 0.47) on a 4-point scale. For the subscales, “shrinking relationships with coworkers” (*M* = 3.10, SD = 0.64) was highest, followed by “overwhelming workload” (*M* = 2.97, SD = 0.62), “confusion in professional values” (*M* = 2.84, SD = 0.65), and “conflict between theory and practice” (*M* = 2.81, SD = 0.54). The mean person-environment fit score was 2.69 (SD = 0.77) on a 5-point scale. The participants perceived that “person-organization fit” (*M* = 2.60, SD = 0.88) was lower than the “person-job fit” (*M* = 2.78, SD = 0.80).

### 3.2. Differences in the Groups' Characteristics

Compared with the turnover group, those in the retention group were statistically significantly younger (*M* = 24.7, SD = 1.2; *p* = 0.18), with higher scores for clinical problem-solving (*t* = 2.029, *p* = 0.044) and professional identity (*t* = 2.230, *p* = 0.027) on the readiness for practice scale. Simultaneously, they had lower transition shock (*t* = −4.978, *p* < 0.001) and higher person-environment fit (*t* = 3.861, *p* < 0.001) scores for all subdomains (Table 1).

### 3.3. CART Analysis for New Graduate Nurses' Retention

**Figure 3:**
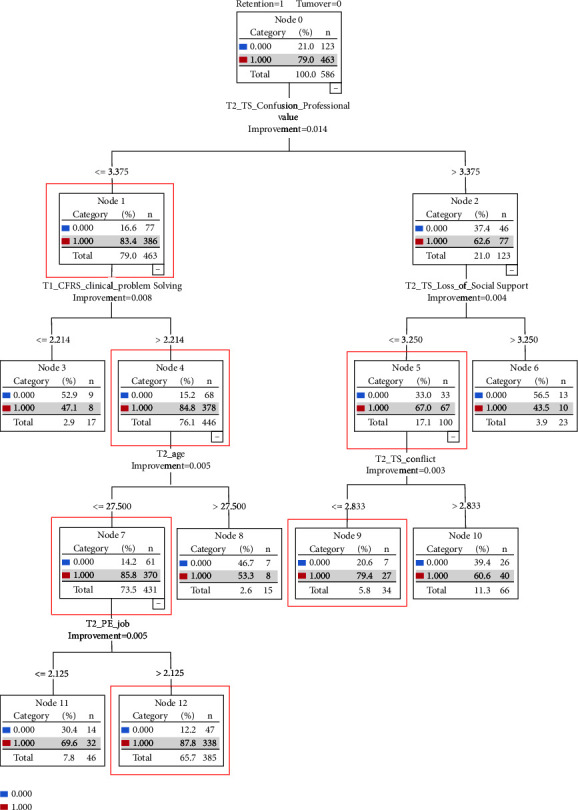
Classification decision tree analysis of the relationship between students and new nurses' characteristics.

The variables (*p* < 0.05) from the univariate analysis were entered into the CART model.  Figure 3 depicts the final decision tree analysis model for predicting new graduate nurse retention, ultimately generating five layers and seven nodes. Based on the conceptual framework [28], in terms of anticipatory socialization, readiness for practice and age were significant; for organizational socialization, transition shock and person-environment fit were substantial. The most important discriminating factors were as follows in the descending order: (sub) confusion in professional values of transition shock, (sub) clinical problem-solving of readiness for practice, age, (sub) person-job fit of person-environment fit, (sub) loss of social support of transition shock, and (sub) conflict between theory and practice of transition shock. Ten groups were identified, six of which had higher percentages of retention than the root node (boxes with red lines in Figure 3). Newly graduated nurses who scored lower than 3.38 for confusion in professional values and higher than 2.21 on clinical problem-solving were younger than 27.5 years; those who scored higher than 2.13 on person-job fit showed a higher retention rate. Otherwise, among the newly graduated nurses, those who scored higher than 3.38 on confusion in professional values, higher than 3.25 on loss of social support and lower than 2.83 on conflict between theory and practice showed higher retention.

We evaluated the quality of this model's performance using the receiver operating characteristic curve, which yielded an area under the curve of 0.700 (95% confidence interval of 0.636–0.750) and accuracy of 79.7%. This indicated acceptable diagnostic accuracy (exceeding 0.7) [35].

## 4. Discussion

To the best of our knowledge, this is the first study to identify educational factors from among school and organizational socialization factors in the workplace associated with the retention of new graduate nurses based on Scott's transition model [28]. Previous studies have found that the organizational-level working environment and personal-level transition shock are predominant factors influencing new nurses' turnover intentions [6, 11, 12]. Our study contributes to existing knowledge by examining the relationship between anticipatory (readiness for practice) and organizational (transition shock and person-environment fit) socialization, which are factors that affect actual turnover and retention. Compared to previous research, using decision tree methods provided an opportunity to review a wide range of factors affecting retention and also revealed the logic behind a retention or turnover decision. The CART model shows that retention is mainly related to the younger age group, higher readiness for practice (clinical problem-solving) in the nursing program, lower transition shock (confusion in professional values, loss of social support, and conflict between theory and practice), and higher person-environment fit (person-job fit).

In this study, approximately 21% of the newly graduated nurses reported that they had experienced job turnover since starting work at a hospital after graduation. According to a recent nationwide study in South Korea, the turnover rate of early career nurses with under three years of experience was 26.7% [36]. Another study using longitudinal panel data reported that 25% of new graduate nurses in South Korea left their jobs within the first year [37]. Considering that the average length of experience for our participants was approximately eight months, these results are consistent with our study. Considering South Korean hospitals generally provide an average of three to six months of training [38], new nurses who turn over within a year will have about six months of clinical experience. The quick turnover of new nurses necessitates more frequent recruitment processes, which cause additional financial burdens for staffing and training and hamper patient outcomes [36, 37].

Considering that the CART model ranked the features based on the Gini coefficient [34], confusion in professional values (a subdomain of transition shock) was ranked first. Our results are consistent with the findings of previous studies on nursing students [39, 40] and nurses [41, 42] indicating that higher professional values result in lower turnover intention. Feng and Tsai [43] found that when conflict occurs between the organizational value of pursuing work-centered nursing and professional value of pursuing patient-centered nursing, new nurses experience high levels of stress and confusion regarding professionalism. These results suggest that advancement of professional values may increase the retention of new nurses by raising their sense of professionalism [41]. Based on these results, professional values can be claimed as a crucial factor in the retention of new nurses [44].

An interesting finding is that the participants who had confidence in clinical problem-solving when they were nursing students tended to remain in their jobs as new nurses. Problem-solving skills are a core competency of professional nurses, who need to continuously identify patients' health problems and make clinical judgments in patient care [45, 46]. Nurse educators should pay attention to the problem-solving ability of new graduate nurses during the transition, optimize the new nurse orientation program, and conduct a series of training sessions using situational simulation and new media technology to improve their clinical reasoning [46].

Regarding age, this study's findings are contrary to those of previous studies that found that young nurses were more inclined to leave their work [10, 36, 47, 48]. According to Bae et al. [36], life events, including pregnancy and childbirth, increase actual turnover among nurses aged 20–35 years. A possible reason for this is the characteristics of our sample. Unlike previous studies, our study included only new graduate nurses in the 22–29-year age range, who had worked less than a year and were still in transition. Previous studies have reported that the age of senior nursing students is negatively correlated with their learning outcomes; confidence in managing multiple patients [49] and nursing skill competency [46] decrease as age increases. Considering that confidence and competence on the learning curve are likely to grow with experience [50], we cannot confirm the effect of age on the retention of nurses. This is because we followed up within a year of graduation, which is a very early career stage with an expectedly unstable transition process. Further studies are required to verify the relationship between age and retention over time.

Another noteworthy finding of this study is the positive relationship between person-job fit and retention, which affects the turnover of new graduate nurses. The more suitable the person-job fit, the more likely it is that new nurses will remain. Person-environment fit has been repeatedly identified as a predictor of turnover or turnover intention in the literature [51, 52]. It is possible that a good fit between the job and organization leads to higher job satisfaction, empowerment, and work engagement [51, 53, 54]. Person-job fit occurs when the staff's knowledge, skills, and abilities match work demands and the work performed meets their needs [55]. This suggests that hospital administrators and nurse managers should consider nurses' characteristics, needs, and values before assigning work and departments to enable them to perform to their full potential. In addition, they should provide nurses with precise and detailed job descriptions, and educational opportunities for updating skills and knowledge to continuously improve their work abilities.

In addition, participants with relatively low professional values were influenced by social support. This finding indicates that even if professional values are not strong, there is a high possibility of retention if strong social support is received. Social support is a job resource that facilitates employees' work motivation, involvement, and engagement, promoting wellbeing [56]. Previous studies have reported that well-supported nurses in hospitals show a higher intention to stay [11, 22]. Therefore, to retain new nurses, who face difficulties with unfamiliar work, unskilled interpersonal relationships, and the unestablished value of their profession [7], nurse managers, coworkers, and supervisors should pay more attention to them at the organizational level. Support activities, such as peer support, collegiality fostering programs, and mentorship, can help new nurses alleviate job stress and promote personal meaningfulness and health and wellbeing at work and ultimately help them find the professional value [57, 58].

This study has several limitations. Despite the internal validation through CART analysis demonstrating excellent accuracy, the generalizability of the model would benefit from external validation using additional databases from different settings or populations. In addition, using convenience sampling resulted in a sample that was predominantly female and consisted of college graduates. This may lead to a lack of diversity. To address this limitation, future research should focus on employing more representative sampling methods, such as random or stratified sampling, to ensure a more diverse and inclusive participant pool. Furthermore, the variables in this study were measured using self-report, which may introduce potential biases or inaccuracies. To improve the robustness of future studies, it is recommended that readiness for practice and organizational socialization be assessed by third-party sources such as nursing faculty, preceptors, or supervisors. This approach provides a more objective and comprehensive evaluation of these factors, thereby enhancing the reliability and validity of the research findings.

## 5. Conclusions

We developed an internally validated decision tree model based on six factors to predict new graduate nurse retention using longitudinal data. This was accomplished by applying a conceptual framework with Scott's transition model for new graduate nurses in the workplace. The prediction algorithms and results of this study will contribute to transforming the educational strategies of undergraduate nursing programs and management policies of hospitals to enhance the retention of new graduate nurses. To retain new nurses in practice settings, nursing educators in schools and managers in hospitals should cooperate to prepare nursing students for practice, provide support to experience success in the organizational socialization process, and cultivate healthy professional values to grow as competent nurses in the workplace.

## Figures and Tables

**Table 1:** Differences in general characteristics between the retention and turnover groups (*N* = 586).

Variables	Categories	Total (586)	Retention (*n* = 463)	Turnover (*n* = 123)	*x* ^2^ or *t* (*p*)
		Mean ± SD or *n* (%)	Mean ± SD or *n* (%)	Mean ± SD or *n* (%)	
*Anticipatory socialization*					
Personal factors					
Age (years)		24.8 ± 1.3 (22–29)	24.7 ± 1.2	25.1 ± 1.6	−2.371 (0.018)
Gender	Women	533 (91.0)	421 (79.0)	112 (21.0)	0.002 (0.965)
	Men	53 (9.0)	42 (79.2)	11 (20.8)	
Educational factors					
Type of school	University	484 (82.6)	383 (79.1)	101 (20.9)	0.025 (0.874)
	College	102 (17.4)	80 (78.4)	22 (21.6)	
Location of school	Capital area	308 (52.6)	237 (76.9)	71 (23.1)	1.655 (0.197)
	Rural	278 (47.4)	226 (81.3)	52 (18.7)	
Second degree	Yes	25 (4.3)	19 (76.0)	6 (24.0)	0.025 (0.874)
	No	561 (95.7)	444 (79.1)	117 (20.9)	
Academic resource availability	Yes	400 (68.3)	315 (78.8)	85 (21.2)	0.051 (0.821)
	No	186 (31.7)	148 (79.6)	38 (20.4)	
Simulation education	Yes	572 (97.6)	452 (79.0)	120 (21.0)	0.002 (0.967)
	No	14 (2.4)	11 (78.6)	3 (21.4)	
Internship	Yes	51 (8.7)	43 (84.3)	8 (15.7)	0.947 (0.330)
	No	535 (91.3)	420 (78.5)	115 (21.5)	
Readiness for practice		2.86 ± 0.31	2.87 ± 0.31	2.80 ± 0.31	2.205 (0.028)
Clinical problem-solving		2.88 ± 0.77	2.90 ± 0.37	2.82 ± 0.38	2.029 (0.044)
Learning technique		2.92 ± 0.55	2.92 ± 0.56	2.92 ± 0.52	−0.045 (0.964)
Professional identity		2.94 ± 0.43	2.97 ± 0.42	2.86 ± 0.47	2.230 (0.027)
Trials and tribulations		2.73 ± 0.35	2.74 ± 0.36	2.69 ± 0.32	1.597 (0.112)

*Organizational socialization*					
Work characteristics					
Work experience in current hospital (Months)		8.3 ± 3.9 (0.03–15.0)	9.4 ± 3.3	4.21 ± 3.0	16.804 (*p* < 0.001)
Working unit	General ward	321 (54.8)	246 (76.6)	75 (23.4)	2.621 (0.270)
	Special care unit (ICU, ER, OR)	237 (33.3)	195 (82.3)	42 (17.7)	
	Others	28 (4.8)	22 (78.6)	6 (21.4)	
Desired unit	Yes	335 (57.4)	272 (81.2)	63 (18.8)	1.751(0.186)
	No	249 (42.6)	191 (76.7)	58 (23.3)	
Number of preceptors	1	366 (62.7)	289 (79.0)	77 (21.0)	0.422 (0.810)
	2	108 (18.5)	88 (81.5)	20 (18.5)	
	Over 3	110 (18.8)	86 (78.2)	24 (21.8)	
Transition shock		2.82 ± 0.47	2.77 ± 0.45	3.00 ± 0.50	−4.978 (*p* < 0.001)
Conflict between theory and practice		2.81 ± 0.54	2.78 ± 0.52	2.94 ± 0.60	−2.821 (0.005)
Overwhelming workload		2.97 ± 0.62	2.93 ± 0.61	3.09 ± 0.66	−2.526 (0.012)
Loss of social support		2.17 ± 0.73	2.12 ± 0.70	2.37 ± 0.79	−3.481 (0.001)
Shrinking relationships with coworkers		3.10 ± 0.64	3.05 ± 0.62	3.28 ± 0.69	−3.497 (0.001)
Confusion in professional values		2.84 ± 0.65	2.77 ± 0.64	3.10 ± 0.62	−5.063 (*p* < 0.001)
Incongruity in work and personal life		2.72 ± 0.78	2.65 ± 0.75	2.97 ± 0.81	−4.042 (*p* < 0.001)
Person-environment fit		2.69 ± 0.77	2.75 ±0 .075	2.45 ± 0.81	3.861 (*p* < 0.001)
Person-job fit		2.78 ± 0.80	2.85 ± 0.77	2.55 ± 0.88	3.748 (*p* < 0.001)
Person-organization fit		2.60 ± 0.88	2.66 ± 0.87	2.36 ± 0.90	3.364 (0.001)

## Data Availability

To ensure the confidentiality of the participants' personal information, the datasets are not publicly available.
